# A real-world analysis of pharmacotherapy adherence and the factors influencing it in Serbia: a nationwide, population-based, cross-sectional study

**DOI:** 10.3389/fpubh.2024.1437796

**Published:** 2024-08-01

**Authors:** Dragana Srebro, Zoran Bukumirić, Milena Šantrić Milićević

**Affiliations:** ^1^Faculty of Medicine, Institute of Pharmacology, Clinical Pharmacology and Toxicology, University of Belgrade, Belgrade, Serbia; ^2^Faculty of Medicine, Institute of Medical Statistics and Informatics, University of Belgrade, Belgrade, Serbia; ^3^Faculty of Medicine, Institute of Social Medicine, University of Belgrade, Belgrade, Serbia; ^4^Faculty of Medicine, Laboratory for Strengthening the Capacity and Performance of Health Systems and Health Workforce for Health Equity, University of Belgrade, Belgrade, Serbia

**Keywords:** compliance, adults, population study, chronic disease, pharmacotherapy, medication intake

## Abstract

**Introduction:**

Monitoring the pharmacotherapy adherence in society is crucial for identifying occurance and causes of potential inadequate use of drugs and inform providers about the need for better customer counceling. It is necessary component of the strategic planning of the quality of healthcare services. This population- based study aimed to assess the medication intake adherence in the Republic of Serbia and the individual factors and health system variables influencing its pattern.

**Methods:**

We applied a cross-sectional approach to study medication intake adherence using a secondary analysis of the latest 2019 Serbian National Health Survey data. The statistical modeling of the pharmacotherapy adherence incorporated sociodemographic data, self-reported disease, and lifestyle behavior.

**Results:**

In 2019, in the representative sample of 12,066 adults in Serbia, requiring prescribed medicine, 49.8% did comply with the prescribed drugs, and 50.2% do not. Participants who adhered to prescribed medication were significantly (*p* < 0.001) older (62.4 ± 14 years), predominantly female (55.3%), had secondary education (48.5%), resided in southern and eastern parts of Serbia (55.5%), and belonged to the lowest income quintile (21.4%). The participants most often take prescribed drugs for hypertension (64.1%) and lower back pain (30.5%), while around 20% take medication for coronary disease, diabetes mellitus, and high blood cholesterol. About 85–92% of participants with financial or general difficulties using prescribed medication.

**Conclusion:**

There is poor medication intake adherence to prescribed medication in Serbia. Gender, age, and region determine the adherence. Also, health-related and healthcare system-related factors impact the use of prescribed medication. Study findings can inform planning the counceling interventions in the target groups where improving medication adherence is necessary, as well as to enhance training of healthcare providers about pharmacotherapy adherence.

## Introduction

Pharmacotherapy adherence refers to the extent to which a patient’s behavior in taking medication aligns with a doctor’s advice regarding timing, dosage, and frequency of use (1, 2). According to the International Society for Pharmacoeconomics and Outcome Research (ISPOR) Medication Compliance and Persistence Working Group, adherence is a synonym for compliance (2). Full adherence is when a patient follows a doctor’s instructions completely. Although opinions vary, non-adherence is generally defined as taking medications less than 80% of the time (3, 4). Consequently, the incidence of suboptimal adherence may vary based on different definitions. In our study, the prevalence with 95% CI of medication intake adherence was studied not among the patients but at the population level.

Healthcare providers may have difficulties in routine control of adherence with prescribed therapy due to various reasons including of patients’ absenteism on sheduled follow-ups, loss of memory among older adult, multimorbidity induced polypharmacy, etc. Non-adherence to the medication can be intentional or unintentional. In unintentional non-adherence, patients typically forget to take their medication, or were unaware of its relevance. With regard to adherence of prescribed generic drugs, which are often less expensive then branded drugs, many authors found out that among demographic, social, psychological and clinical factors a lower income of patients is significant for optimal adherence, regardless of their gender or age (5). There are many reasons for intentional non-adherence, including experiencing or anticipating side effects, disagreement with the pharmacological treatment, and an inability to pay for the medications (6). According to data from the World Health Organization, about 50% of patients with chronic diseases do not consistently follow the treatment recommendations of pharmacists and doctors, resulting in a lack of the expected response to proven effective medications (7). It is estimated that up to 30% of prescribed drugs never reach the pharmacist for dispensation (3). As a result of insufficient patient adherence, treatment success is questioned, leading to unwanted quality of life and clinical outcomes, unnecessary healthcare utilization and treatment costs, which ultimately may additionally strain the healthcare budget (8–10). It has been estimated that in the US, of all medication-related hospital admissions, about 33–70% result from poor adherence, which represents an annual cost of US$ 100 billion (11).

Adherence can be measured by subjective (patient self-reports, family interviews, and questionnaires) and objective methods (prescription refill rates, residual pill counting, and electronic measurement devices), and there is no specific measure of medication adherence. In our study focus was on self-reports of pharmacotherapy adherence, which allowed us also exploring the respondents’ awareness and reasons of the applied adherence patterns.

Many studies explore medication adherence for specific disease (12–14), factors (15), or drugs (16), and most of these investigations are within specific healthcare institutions or regions (13, 15). In Serbia, patients with Parkinson’s disease and depression compared to Parkinson’s disease patients without depression have a three times higher risk for lower adherence to pharmacotherapy (12). A recently published study conducted in one of the larger cities in Serbia showed that adherence to hypertension medications is poor and that non-adherence is more common when taking more medications and a more complicated dosing regimen, and among residents of the urban settlements (13). Additionally, in Serbia, one study has investigated self-medication (the use of medicine without a doctor’s prescription) at the population level (17). The prevalence of self-medication was 27.1%, and was associated with sociodemographic and health-related factors, as well as unmet healthcare needs (17). To the best of our knowledge, no studies have assessed nationwide medication intake adherence to prescribed medication within the Serbian population.

This study aimed to assess the prevalence of medication intake adherence to prescribed medication among adults in Serbia and identify factors that might affect adherence. We focused on examining the impact of social and demographic factors, disease-related factors, and barriers within the healthcare system.

## Methods

### Study design

This is a population-based, cross-sectional study. The study is a secondary analysis of the Serbian National Health Survey data conducted between October and December 2019 by the Institute of Statistics of the Republic of Serbia in cooperation with the Institute of Public Health of Serbia “Dr. Milan Jovanovic Batut” and the Ministry of Health of the Republic of Serbia.

The study followed the Declaration of Helsinki (1964 and 2013) and was approved by the Ethics Committee of Faculty of Medicine University of Belgrade, Belgrade, Serbia (permit number 1322/IX-9). To safeguard the privacy of survey participants and collected data, all procedures were in accordance with the General Data Protection Regulation and the National Personal Data Protection Act.

### Sample

Subject sampling and the study protocol were implemented in accordance with the guidelines of the European Health Interview Survey-third wave (EHIS wave 3) (18). In brief, a two-stage stratified sample was used based on the type of settlement (urban or other) and geographical areas (four statistical regions: Vojvodina, Belgrade, Šumadija, and western Serbia, southern and eastern Serbia). The survey included a representative sample size of the population, based on Eurostat recommendations for conducting population health surveys. From 13,589 participants, based to the focus of this research and after adjust for age (18 years and older) and answers from the use of drugs prescribed by a doctor (yes/no) the total number of participants was 12,066.

### Research instrument and variables and setting

For data collection, a face-to-face self-reported questionnaire was used. The questionnaire was standardized and based on Eurostat (19). Trained interviewers gave the participants a printed document with information about the survey.

The sociodemographic characteristics, health status (disease and chronic conditions), use of prescribed medicines by participants, and information regarding the healthcare system were extracted from the National Health Survey 2019 database. The following specific questions about regular use of the medication were used: ``*During the past two weeks, have you used any drugs prescribed by your doctor?*`` (yes or no), ``*Do you have difficulty performing certain activities - taking medication?*`` (yes or no) and ``*In the past 12 months, have you needed prescribed medication but could not afford it due to financial reasons?*`` (yes or no).

The variable ``*During the past two weeks, have you used any drugs prescribed by your doctor?``* was dichotomized into a variable with two modalities, “no” and “yes,” where “yes” was the reference category and represented the dependent variable. That variable (medication intake adherence) is related to the use of drugs prescribed by a doctor (relevant to study the adherence, as proposed by EHIS wave 3 Methodological Manual instructions) served as the basis for creating the database in this study. The analysis included pharmaceutical drugs, while vitamins, minerals, herbal medicines, and herbal teas (unless classified as medicines) were excluded. The analysis included the following independent variables: (1) sociodemographic (age years as continuous variable 18+), gender (male/female), level of education (no formal education/primary school/secondary school/ undergraduate studies/postgraduate studies), socioeconomic status (income quintile, five categories from lowest to highest); (2) health-related including the presence of a chronic disease from a list of most common diseases in the population (yes/no). The following question was used*:``Have you had any of the above diseases or conditions in the past 12 months?``* with answers yes or no; (3) healthcare system-related, such as unmet healthcare needs (medication intake) in the past 12 months due to either inability to afford prescribed medication for financial reasons, or difficulty in taking medication. The following questions were used: *``In the past 12 months, have you needed prescribed medication but could not afford it due to financial reasons?``* with answers yes or no and *``Do you have difficulty performing certain activities - taking medication?``* with answers yes or no.

The outcome of interest was medication intake adherence to prescribed medication. In most studies, adherence is defined as taking 80% or more of the prescribed medication doses (3, 4). However, depending on the outcome, that threshold of 80% can be different and cannot be confirmed or rejected (20). It is estimated that adherence to chronic medications is around 50%. Based on the literature data, adherence to medication is considered low or suboptimal if greater than 20–50% of participants are non-adherent to prescribed medications (12, 21–25).

### Statistical analysis

The data are presented with descriptive statistics as n (%), arithmetic mean ± standard deviation or median (min-max), depending on the type of variables and the normality of the distribution. The t- and chi-square tests were used to test statistical hypotheses. Logistic regression was used to model the relationship between the dependent variable (medication intake adherence with prescribed pharmacotherapy) and potential predictors. Variables significantly associated with the prescribed drugs in the univariate analysis were included in the multivariate logistic regression models. Statistical significance in all analyses was considered if the computed probability value was ≤0.05. The results are presented in tabular and graphic form. All data were processed in the IBM SPSS Statistics 24 (SPSS Inc., Chicago, IL, United States) software package or the R programming environment.

## Results

### Adherence with prescribed pharmacotherapy

The final total sample included 12,066 adult participants requiring medicine 2 weeks before survey. The prevalence of prescribed medicine use is 49.8% (95% CI 48.9–50.1). Participants who used and did not use prescribed medications in the previous 2 weeks were almost equally distributed in the study sample, 6,005 (49.8%), and 6,061 (50.2%), respectively (Table 1).

**Table 1:** The use of prescribed medications and sociodemographic characteristics.

Variable	Total *n* (%)	Yes *n* (%)	No *n* (%)	*p*-value
**Use of prescribed medications in the previous 2 weeks**	12,066	6,005 (49.8%)	6,061 (50.2%)	
**Gender**, *n* (%) Male Female	12,066 5,834 (48.4%) 6,232 (51.6%)	2,558 (43.8%) 3,447 (55.3%)	3,276 (56.2%) 2,785 (44.7%)	<0.001
**Age**, median, min-max		64 (18–99)	40 (18–99)	<0.001
**Region** Belgrade Region Vojvodina Region Šumadija and western Serbia Southern and eastern Serbia	12,066 2,847 (23.6%) 2,665 (22.1%) 3,934 (32.6%) 2,620 (21.7%)	6,005 (49.8%) 1,294 (45.5%) 1,403 (52.6%) 1853 (47.1%) 1,455 (55.5%)	6,061 (50.2%) 1,553 (54.5%) 1,262 (47.4%) 2081 (52.9%) 1,165 (44.5%)	<0.001
**Education level** No formal education Primary school Secondary school Undergraduate (vocational studies) Undergraduate (academic studies) Postgraduate studies (MSc, PhD)	12,062 924 (7.7%) 2030 (16.8%) 6,736 (55.8%) 889 (7.4%) 1,243 (10%) 240 (2%)	6,003 (49.8) 731 (12.2%) 1,322 (22%) 2,912 (48.5%) 46 (7.7%) 482 (8%) 89 (1.5%)	6,059 (50.2%) 193 (3.2%) 708 (11.7%) 3,824 (63.1%) 422 (7%) 761 (12.6%) 151 (2.5%)	<0.001
**Socioeconomic status** 1- lowest income quintile 2 3 4 5 – highest income quintile	12,066 2,432 (20.2%) 2,437 (20.2%) 2,471 (20.5%) 2,434 (20.2%) 2,292 (19.0%)	6,005 (49.8%) 1,287 (21.4%) 1,265 (21.1%) 1,230 (20.5%) 1,176 (19.6%) 1,047 (17.4%)	6,061(50.2%) 1,145 (18.9%) 1,172 (19.3%) 1,241 (20.5%) 1,258 (20.8%) 1,245 (20.5%)	<0.001

### Adherence and association with sociodemographic characteristics

Participants who adhered, take prescribed medication in the last 2 weeks, were statistically significant (*p* < 0.001), predominantly female (55.3%), had secondary education (48.5%), resided in southern and eastern Serbia (55.5%), and belonged to the lowest income quintile (21.4%). Older participants (average age 62.4 ± 14) used prescribed drugs more often than younger participants (average age 41.8 ± 15.5). Participants who not take the prescribed medication were predominantly men, participants with secondary school and higher income quintile, resided in Belgrade region. Detailed sociodemographic characteristics of the study sample are shown in Table 1.

### Use of prescribed medications associated with disease

The use of prescribed medicines for certain diseases is significantly (*p* < 0.001) higher than non-use. Participants who reported taking prescribed medicines significantly (*p* < 0.001) suffered from hypertension (64.1%) and lower back pain (30.5%), while about 20% of the respondents had coronary disease, cervical spine pain, diabetes mellitus and high blood cholesterol (Table 2). Our results indicated that there are probably respondents with more than one specific disease, since that the total number of respondents who have specific disease (16.187) is higher than total number of participants (12.066) (Table 2).

**Table 2:** Use of prescribed medications and presence of disease.

Variable	Total *n* (%)	Yes *n* (%)	No *n* (%)	*p*-value
Use of prescribed medications in the previous 2 weeks	12,066	6,005 (49.8%)	6,061 (50.2%)	
Asthma	462 (3.8%)	394 (6.6%)	68 (1.1%)	<0.001
COPD*	447 (3.7%)	388 (6.5%)	59 (1.0%)	<0.001
AIM*	255 (2.1%)	244 (4.1%)	11 (0.2%)	<0.001
Coronary disease	1,240 (10.3%)	1,192 (19.9%)	48 (0.8%)	<0.001
Hypertension	4,100 (34%)	3,849 (64.1%)	251 (4.2%)	<0.001
Stroke	160 (1.3%)	153 (2.5%)	7 (0.1%)	<0.001
Arthrosis	971 (8.1%)	860 (14.3%)	111 (1.8%)	<0.001
Lower back pain	2,318 (19.2%)	1833 (30.5%)	485 (8%)	<0.001
Cervical spine pain	1,633 (13.5%)	1,317 (21.9%)	316 (5.2%)	<0.001
Diabetes mellitus	1,087 (9%)	1,049 (17.5%)	38 (0.6%)	<0.001
Allergy	863 (7.2%)	579 (9.6%)	284 (4.7%)	<0.001
Inability to hold urine	450 (3.7%)	405 (6.7)	45 (0.7%)	<0.001
Depression	548 (4.5%)	492 (8.2%)	56 (0.9%)	<0.001
High blood cholesterol	1,428 (11.9%)	1,287 (21.6%)	141 (2.3%)	<0.001
Cancer	225 (1.9%)	202 (3.4%)	23 (0.4%)	<0.001

*COPD, chronic obstructive pulmonary disease; AIM, acute myocardial infarction.

### Adherence with prescribed pharmacotherapy associated with the healthcare system

Analysis showed that prescribed medication use was significantly (*p* < 0.001) associated with unmet healthcare needs in the past 12 months (Table 3). There is a statistically significant (*p* < 0.001) difference in the frequency of respondents who have financial difficulties in obtaining medication (728/12050) in relation to whether or not the respondents take prescribed medicine (Table 3). However, the respondents with financial difficult to afford medications still in a high extent (85%) intake prescribed medications. The respondents with financial difficulties were more prevalent in the Belgrade region, as well as eastern and southern Serbia. There is a statistically significant difference (*p* < 0.001) in the frequency of difficulty taking medication in relation to whether or not the respondents are taking the prescribed medication. About 91% of respondents who used medication typically had difficulty (minor, major difficulties or total incapacity) in taking prescribed medication (Table 3). Difficulties in performing daily activities, particularly in adhering to therapy (medicines), are significantly (*p* < 0.001) higher in older people (78 ± 8–9 years old), women compared to men (62% vs. 37.7%), and in the regions of Šumadija, western, southern, and eastern Serbia.

**Table 3:** Use of prescribed medications and healthcare system related characteristics.

Variable	Total *n* (%)	Yes *n* (%)	No *n* (%)	*p*-value
Difficulty taking medication	335/5675 (5.9%)	307 (91.6%)	28 (8.4%)	<0.001
Cannot afford the medicine for financial reasons – in the last 12 months	728/12050 (6%)	622 (85.4%)	106 (14.6%)	<0.001

### Predictors of adherence with prescribed pharmacotherapy

In a multivariate logistic regression model, medication intake adherence with prescribed pharmacotherapy was the dependent variable, while social and demographic independent variables were included as adjusted for the existence of a disease or condition in the last 12 months. There was no significant multicollinearity between the social and demographic variables.

**Figure 1:**
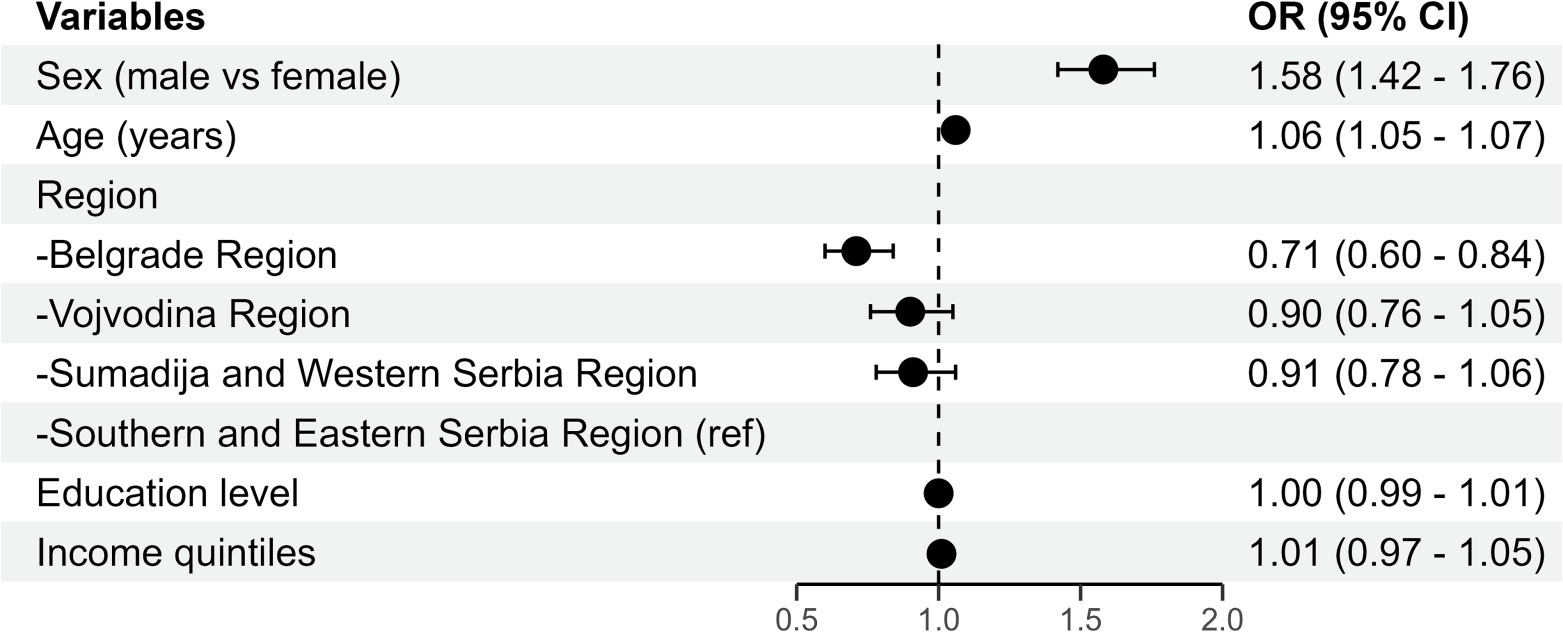
Forest plots with odds ratios (ORs) and associated confidence intervals (CIs) (95%) of the statistically significant results of multivariate analysis.

In the multivariate logistic regression model, statistically significant potential predictors of medication intake adherence with prescribed pharmacotherapy were female gender (*B* = 0.460; *p* < 0.001), older age (*B* = 0.060; *p* < 0.001), and the region [Belgrade (*B* = -0.344; *p* < 0.001)], with southern and eastern Serbia as a reference category (Figure 1).

## Discussion

This was the first study to investigate the characteristics and predictors of prescribed medication usage at the national level in Serbia, allowing to understand the frequency and reasons for non- adherence with prescribed pharmacotherapy. The prevalence of prescribed medicine use is 49.8% (95% CI 48.9–50.1). The study revealed a significantly high frequency (about 50%) of non-use of prescribed medicines in the population and poor adherence. Significant associations between the sociodemographic factors and prescribed medication use among adults in Serbia were observed, as well as the impact of health- and healthcare system-related factors and prescribed medication use. Based on these findings, gender, age, and region are factors determining the use of prescribed medication in Serbia. Among the adult population, female gender, older age, and residence in the regions of southern and eastern Serbia are significantly associated with higher use of prescribed medicines. Also, individuals with secondary school education and belonging to the lowest income quintile were not associated with medication intake adherence to the prescribed medication. In Serbia, the population primarily takes prescribed drugs to treat conditions such as hypertension, lower back pain, coronary disease, cervical spine pain, diabetes mellitus, and high blood cholesterol. Our results indicate that the population have unmet healthcare needs due to financial constraints or difficulties in adhering to medication therapy which significantly affect the use of medicines, but on the other hand people have high prevalence (85–92%) of medication intake adherence to prescribed medications.

Between the two national surveys, a slight increase in the frequency of prescribed drug use in the population was observed. In 2013, about 44% of the population reported using medicines prescribed by a doctor within the 2 weeks before the survey (26); results of this study show that this frequency was 49% in 2019. However, our results indicate that around 50% of the participants did not use prescribed medicines, which points a high prevalence of nonintake of medication and to poor adherence. Since 98% of the population has obligatory social health insurance, in the Serbian predominant Bismarck social model (income-based and financed jointly by employers) of health system, a burden of costs for prescribed pharmacotherapy is shared between the Health Insurance Fund (27) and out-of-pocket copayment. In 2013, female gender, middle age, and chronic disease were sociodemographic factors associated with self-medication in Serbia (17). Similarly, in our study conducted in 2019, these same factors were found to be associated with the adherence with prescribed pharmacotherapy. As this is about the use of prescribed and non-prescribed drugs, it is possible that a certain degree of self-medication is also present in our respondents.

Our results are in the accordance with the results from population-based study in Germany (21). In Germany at least 33% of population does not use prescribed medication and nonadherence to medication occurs more often in younger populations with higher socioeconomic status than in older populations with chronic conditions (21). Results of the National Health Survey in Spain showed that about 92% of participants take some medication prescribed by a doctor, but unlike Serbia, they included only respondents aged 65+ which does not allow the comparisons (28). For instance, in our research, the age was important from two distinct perspectives. Middle aged individuals (about 40 years and older) were more prone to be non-adherent, and older individuals (about 78 years and above) had difficulties in medicine use. The majority of participants use prescribed medicines for specific diseases, a factor that positively influences treatment outcomes and health preservation Similar to our results, in Spain women take prescribed drugs significantly more often than men, and these drugs are mostly used for the treatment of hypertension, diabetes, and other chronic diseases (28). Our results agree with data obtained from the adult population in Austria, indicating that individuals from less educated and lower socioeconomic groups were more likely to use prescribed medicines (29). One of the most important predictor of nonadherence may be out-of-pocket costs for medication (30). Our results suggest that although participants have difficulties in taking and buying medications, there is still a high prevalence (85–92%) of the intake of prescribed medication and good adherence in relation to the general intake medication adherence. In some patients (both low-income and high-income, depression and asthma), it is possible that cost associated nonadherence with may be influenced by patient beliefs, about the perceived value of prescription medication (31). In general, it is difficult to directly compare our results to results of other studies due to methodological differences in sample selection, assessment of adherence, type of drugs and disease and differences between countries.

Study about medication intake adherence was performing in Albania in similar period. As in Serbia and in Albania, there is a high incidence of nonintake of prescribed medication (about 20%) in adult population (23). Factors associated with nonintake of medication in Albania included rural residence, low educational level and low economic level, while in Serbia were residence in capital region, lower educational and higher economic level. In both countries, financial difficulties have a significant impact on the use of prescribed medication, however, in Serbia the frequency of medication intake is still high (85%) compared to Albania (9%). As Albania is a country in transition in Southeast Europe, just like Serbia, the results are interesting to compare because both studies included intake medication adherence and associated factors. However, the results cannot be fully compared, given that the study was in Albania included primary users health care, not general population.

The descriptive data on the extent of medication intake (non-)adherence in the Serbian population are highly informative. The present real-world analysis included 12,066 adults (18+ years) who need prescribed medicine and was not limited by selection bias. The study findings can inform stakeholders efforts toward improving medication adherence in the population and enhancing implementation of national healthcare programs in specific population groups. Moreover, a recommendation is to add more specific questions for polipharmacy into the questionnaire for the next National Health Survey. Pharmacoepidemiologic data from the Health Surveys may be limited due to self-reporting if understanding of the adherence with prescribed pharmacotherapy differs among respondents. The suboptimal level of pharmacotherapy adherences indicates a necessity for rising awareness and counseling of its relevance among people as well as its control among pharmacotherapy prescribers.

This study has some limitations. For monitoring the medication adherence we used subjective method - patient interview and self-report assessments. This is simple to use and less expensive method for measure of medication adherence, but respondents may be biased and give expected answers. However, this is a one of the most common tool for measure of medication adherence, especially useful for nationwide study. As the study was conducted in the population and as a part of a national investigation, the results are useful in the future investigations of medication adherence and in acting on critical factors for a patient’s non-adherence and in developing policy to improve adherence in improving treatment outcomes and reducing overall health costs. Next, this survey include medication intake adherence but not determine adherence using another tools. It would be more appropriate, if the survey determine taking medication with more questions and only for specific disease and determine which number taking medication for more than one disease etc. Furthermore, in this study we controlled only the use of prescribed drugs, while the use of drugs on its own was not included. Also, self-medication in these patients can affect the adherence in prescribed pharmacotherapy. In addition language barriers in some regions (e.g., Vojvodina) should be assessed.

## Conclusion

This study gives evidence about the extent and sociodemographic determinants of medication intake adherence among adult population in a transitional South Eastern European region. Our research shows that lower adherence to prescribed medicines is common in the adult population in Serbia. Gender, age and geographic region are the main social and demographic predictors for prescription medicine use. In Serbia, the population most often takes prescribed drugs for the treatment of chronic disease such as hypertension, coronary disease, diabetes mellitus and high blood cholesterol. Although population has difficulties in taking and buying medicines, there is still a high prevalence of the use of prescribed medicines and good adherence in relation to the general intake medication adherence. The priority population groups for improving adherence with prescribed pharmacotherapy includes men, young adults, individuals with higher levels of education and income, and the population residing in the Belgrade region. Study findings can inform for planning health politics because there are determinants that can be influenced by improving health regulations and training health service providers on the necessity of monitoring and advising patients on pharmacotherapy adherence. Decision-makers and policy-makers in Serbia should consider these findings because the costs of noncompliance may be higher.

## Data availability statement

The data supporting the conclusions of this article will be made available by the authors, without undue reservation.

## Ethics statement

Ethical review and approval was not required for the study on human participants in accordance with the local legislation and institutional requirements. Written informed consent from the [patients/ participants OR patients/participants legal guardian/next of kin] was not required to participate in this study in accordance with the national legislation and the institutional requirements.

## Author contributions

DS: Conceptualization, Data curation, Formal analysis, Investigation, Methodology, Supervision, Visualization, Writing – original draft, Writing – review & editing. ZB: Software, Visualization, Writing – review & editing. MŠM: Data curation, Writing – review & editing.
